# A New Baroreceptor Sensitivity-Restoring Ca-Channel Blocker Diminishes Age-Related Morning Blood Pressure Increase in Hypertensive Patients: Open-Label Monitoring of Azelnidipine Treatment for Hypertension in the Early Morning (At-HOME) Study

**DOI:** 10.3390/ph3010225

**Published:** 2010-01-19

**Authors:** Kazuomi Kario, Masayuki Shirayama, Katsutoshi Hiramatsu, Kazuhito Shiosakai, Mitsunori Sugiyama, Kazuyuki Shimada

**Affiliations:** 1 Division of Cardiovascular Medicine, Department of Medicine, Jichi Medical University School of Medicine, 3311-1 Yakushiji, Shimotsuke, Tochigi, 329-0498, Japan; 2 Daiichi Sankyo Company Limited, 3-5-1 Nihonbashi-honcho, Chuo-ku, Tokyo 103-8426, Japan

**Keywords:** ca-channel blocker (CCB), azelnidipine (AZ), morning hypertension, morning-evening blood pressure difference (ME-Dif)

## Abstract

*Background:* Morning blood pressure (BP) surge, which exhibits an age-related increase, is a risk factor for stroke in elderly hypertensive patients, independently of the 24-h BP level. We studied the effect of the new baroreceptor sensitivity (BRS)-restoring Ca-channel blocker (CCB) azelnidipine (AZ) on this age-related morning BP increase. *Methods:* We conducted a 16-week prospective study to clarify the effect of morning dosing of AZ on home BPs measured in the morning and in the evening in 2,546 hypertensive patients (mean age, 65.1 years; female, 53.6%). *Results:* At baseline, ME-Dif (morning systolic BP [SBP]–evening SBP) increased with age, independently of ME-Ave (average of the morning and evening SBPs). This age-related increase of ME-Dif was exaggerated by regular alcohol drinking and beta-blocker use. After AZ treatment (14.3 ± 3.6 mg/day), ME-AV and ME-Dif were significantly reduced independently of each other, with reductions of –18.1 ± 15.6 and –2.5 ± 13.2 mmHg, respectively (both p < 0.001). AZ treatment decreased age-related increase in ME-Dif particularly in patients who were regular consumers of alcohol and in beta-blocker users. *Conclusions:* The new BRS-restoring CCB AZ significantly reduced age-related increase in morning BP and had some potential benefit on cardiovascular protection in hypertension, particularly in elderly patients and/or consumers of alcohol.

## 1. Introduction 

Cardiovascular events tend to occur most frequently in the morning [1], and morning rise in blood pressure (BP) has been reported to increase the risk for cardiovascular mortality in the general population [2]. We previously found that among clinic, 24 h, awake, sleep, evening, pre-awake, and morning systolic BP (SBP), the latter is the strongest predictor for stroke events in elderly hypertensive patients, with a 10 mmHg increase in morning SBP corresponding to a relative risk of 1.44 (p < 0.0001). In addition, we reported that patients whose average morning/evening SBP (ME-Ave) is > 135 mmHg and in whom the difference of morning SBP and evening SBP (ME-Dif) is >20 mmHg exhibit 6.6 times higher relative risk for cerebral infarction compared with normotensive subjects [3]. Nishinaga *et al*. [4] showed in their population-based study that in patients aged >75 years ME-Dif > 15 mmHg and ME-Ave > 135 mmHg together conferred 12.3 times higher risk for loss of functional independence, whereas Ikeda *et al*. [5] reported that morning rise in SBP compared with evening SBP > 10 mmHg is a strong predictor of cardiac hypertrophy independent of age, insulin resistance, and morning SBP level. We previously reported in untreated hypertensive patients that ME-Dif is positively correlated with left ventricular mass index (LVMI) and that arterial stiffness (expressed as home pulse pressure and brachial-ankle pulse wave velocity, baPWV) is a significant determinant of SBP independently of age, sex, and habitual alcohol consumption [6]. Furthermore, ME-Dif is increased with age, use of beta-blockers, and regularly drinking alcohol [7].

**Figure 1:**
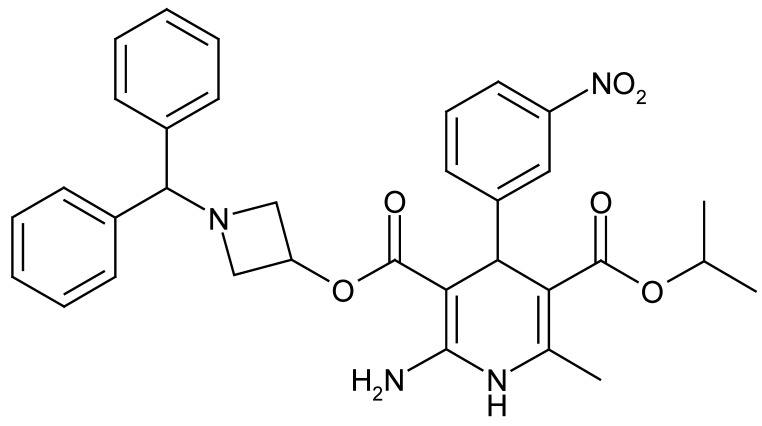
Structure of azelnidipine.

Baroreflex sensitivity (BRS) is a primary mechanism for acute and chronic control of BP. BRS is decreased with age [8,9], and the age-related increase in ME-Dif, one of the measures of BP variability, may be associated with decrease of BRS. Azelnidipine (AZ, Calblock, Figure 1) is a novel long-acting dihydropyridine Ca-channel blocker (CCB) that exerts potent and long-lasting BP-lowering activity for 24 h [9] and reduces heart rate compared with other CCBs such as amlodipine [10,11]. These actions of AZ are considered due to inhibitory effects on sympathetic nervous activity [12]. We also previously demonstrated that AZ ameliorates BRS in hypertensive patients [13].

The present study is a part of the Azelnidipine Treatment for Hypertension Open-label Monitoring in the Early Morning study (At-HOME study) program [14]. The At-HOME study was a 16-week, prospective, observational study conducted in hypertensive patients who received AZ in the setting of daily clinical practice in Japan between May 2006 and May 2007. In the present subanalysis, we looked at data on 2,546 hypertensive patients whose morning BP and evening BP were measured before and after 16 week treatment with AZ, to confirm that ME-Dif is increased with age before dosing of AZ and to investigate the effects of AZ on age-related increase in ME-Dif based on the hypothesis that this agent enhances BRS and inhibits sympathetic nervous activity. Furthermore, we identified modifiable factors potentiating the age-related increase in ME-Dif and investigated whether treatment with AZ exerts beneficial effects in individuals with these risk factors

## 2. Results

### 2.1. Baseline characteristics

Baseline characteristics of patients are summarized in Table 1. Average age was 65.1 ± 11.7 years; female patients accounted for 53.6%. Morning home BP and evening home BP at baseline were 156.9±16.1/89.7±11.7 and 150.2±17.6/85.6±12.2 mmHg, respectively. Pulse rate in the morning and evening was 72.1 ± 10.2 and 72.5 ± 9.6 beats/min. The average initial daily dose of AZ was 13.3 ± 3.9 mg. Morning home BP was determined before breakfast and dosing in 86.8% of patients throughout the observation period.

**Table 1:** Baseline characteristics of patients.

Parameter		Value
Age (years)		65.1 ± 11.7
15–<65, n (%)		1168 (45.9)
65–<75, n (%)		806 (31.7)
75–, n (%)		571 (22.4)
Female (%)		53.6
Body mass index (kg/m^2^)		24.3 ± 3.6
Regular alcohol drinking (almost every day), n (%)		460 (18.1)
Current smoking, n (%)		371 (14.6)
Clinic SBP, mmHg		157.5 ± 18.7
Clinic DBP, mmHg		88.9 ± 13.4
Clinic pulse rate, beats/min		74.8 ± 10.9
Morning SBP, mmHg		156.9 ± 16.1
Morning DBP, mmHg		89.7 ± 11.7
Morning pulse rate, beats/min		72.1 ± 10.2
Evening SBP, mmHg		150.2 ± 17.6
Evening DBP, mmHg		85.6 ± 12.2
Evening pulse rate, beats/min		72.5 ± 9.6
Heart disease, n (%)		305 (12.0)
	Cerebrovascular disease, n (%)	178 (7.0)
	Renal disease, n (%)	106 (4.2)
	Previous antihypertensive medication, n (%)	1407 (55.3)
	Calcium channel blocker	591 (23.2)
	Angiotensin receptor blocker	936 (36.8)
	ACE inhibitor	156 (6.1)
	Diuretic	159 (6.2)
	Alpha-blocker	93 (3.7)
	Beta-blocker	189 (7.4)
	Other	42 (1.6)
	Timing of morning BP measurement, n (%)	
	Before breakfast and AZ dosing	2,209 (86.8)
	Other	337 (13.2)

Abbreviations: ACE, angiotensin-converting enzyme; SBP, systolic blood pressure; DBP, diastolic blood pressure. Values are mean ± SD or n (%).

### 2.2. Time-course of home BP and home pulse rate 

**Figure 2:**
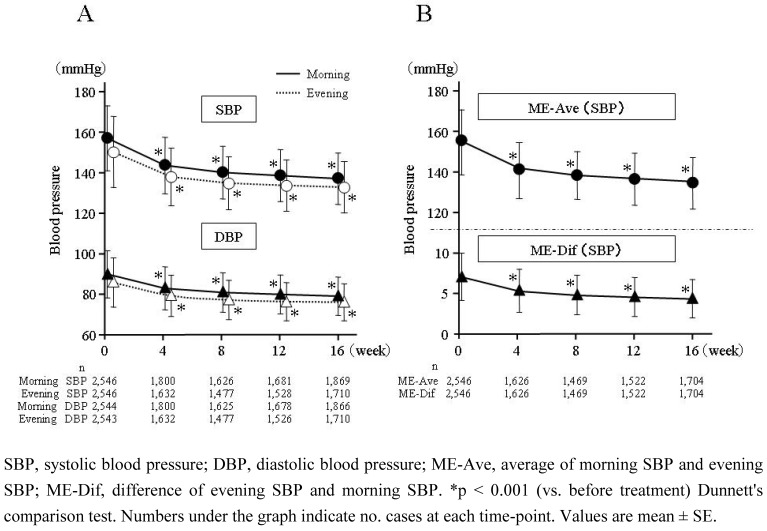
Time-course of change in home morning BP (straight line in A) and evening BP (dotted line in A), ME-Ave (upper graph in B), and ME-Dif (lower graph in B).

Time-courses of average morning home BP and evening home BP are shown in Figure 2A. At baseline, morning home BP and evening home BP were 156.9±16.1/89.7±11.7 (n = 2,546) and 150.2°17.6/85.6°12.2 (n = 2,546) mmHg, respectively, whereas at the end of the treatment period they were 137.6°13.0/79.3°9.7 (n = 2,303) and 133.1°13.0/76.0°9.3 (n = 2,108) mmHg, respectively.

Significant hypotensive effects were detected at four weeks (p < 0.001), and lasted thereafter thru week 16. Change of SBP/DBP from baseline at morning and evening was –19.4±17.1/–10.3±10.6 (n = 2,303) and –16.9±17.0/–9.4±10.6 (n = 2,108) mmHg, respectively (p < 0.001). At baseline, early-morning home pulse rate and evening home pulse rate were 72.1 ± 10.2 (n = 2,213) and 72.5 ± 9.6 (n = 2,190) beats/min, respectively, whereas at the end of the treatment period they were 68.6 ± 9.2 (n = 2,038) and 69.1 ± 8.6 (n = 1,880) beats/min, respectively. Change of pulse rate at morning and evening was –3.5 ± 7.8 (n = 1,972) and –3.5 ± 7.3 (n = 1,833) beats/min, respectively (p < 0.001).

### 2.3. Time-course of ME-Ave and ME-Dif 

The time-course of ME-Ave and ME-Dif during the treatment period is shown in Figure 2B. ME-Ave decreased from 153.8 ± 15.5 mmHg (n = 2,546) at baseline to 135.6 ± 11.9 mmHg (n = 2,408) after the treatment period, a significant (p < 0.001) –18.1 ± 15.6 mmHg (n = 2,101) reduction. Meanwhile, ME-Dif significantly (p < 0.001) decreased by –2.5 ± 13.2 (n = 2,101) from 6.7 ± 13.1 (n = 2,546) to 4.7 ± 10.8 (n = 2,408) mmHg.

### 2.4. Relationship between ME-Dif and age

**Figure 3:**
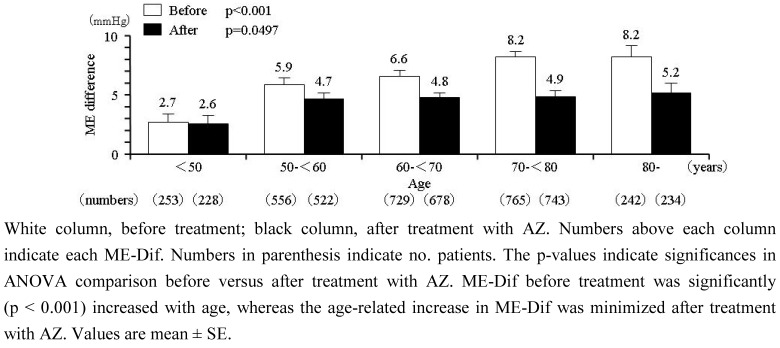
ME-Dif in patients stratified by 10-year age groups.

**Figure 4:**
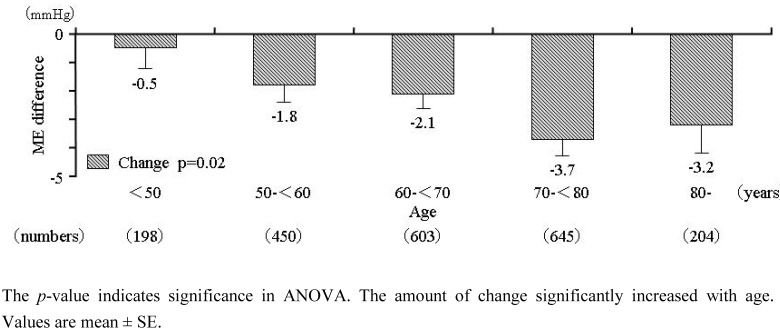
Change of ME-Dif from baseline after treatment with AZ in patients stratified by 10-year age groups.

Regression analysis revealed a strong and significant relation (r = 0.115; p < 0.001) between ME-Dif and patients' age. ME-Dif before and after dosing in patients stratified by age is shown in Figure 3. At baseline, ME-Dif in patients aged < 50, 50–59, 60–69, 70–79, and ° 80 years was 2.7 ± 11.2, 5.9 ± 12.6, 6.6 ± 12.5, 8.2 ± 13.7, and 8.2 ± 14.4 mmHg, respectively, indicating that this parameter increased significantly (p < 0.001) with age. Following AZ treatment, the age-related increase in ME-Dif was suppressed to 2.6 ± 9.1, 4.7 ± 10.0, 4.8 ± 10.7, 4.9 ± 11.5, and 5.2 ± 11.9 mmHg, respectively (p = 0.0497 among groups). Change of ME-Dif in each age group was –0.5 ± 10.3, –1.8 ± 12.5, –2.1 ± 12.8, –3.7 ± 14.5, and –3.2 ± 14.0 mmHg (Figure 4); the change was confirmed significantly (p = 0.02) to increase with age. Average initial doses of AZ in patients aged < 50, 50–59, 60–69, 70–79, and ° 80 years were 13.0 ± 3.9, 13.2 ± 3.9, 13.4 ± 3.9, 13.5 ± 3.8, and 13.2 ± 3.9 mg, respectively. There were no differences of AZ dosage among age groups. 

### 2.5. Determinants of age-related increase in ME-Dif 

**Figure 5:**
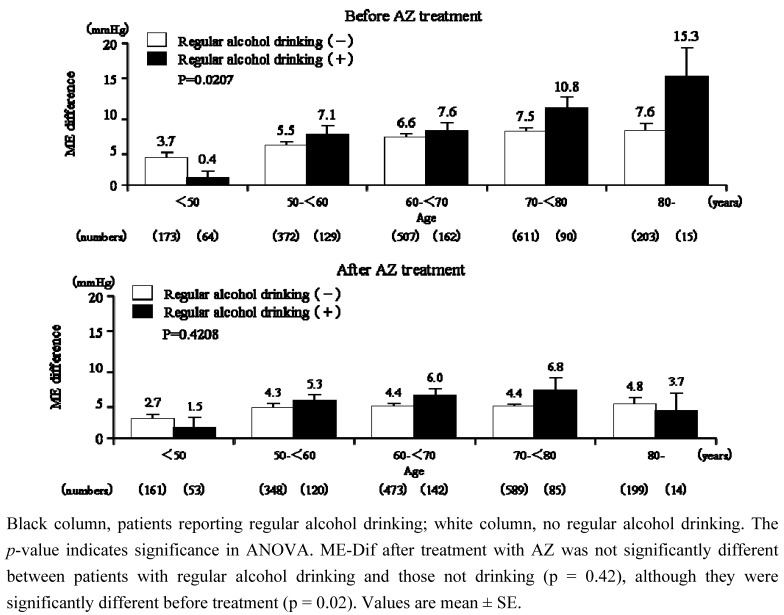
ME-Dif in patients with or without regular alcohol drinking stratified according to age before and after treatment with AZ.

Although beta-blocker use and regular alcohol drinking significantly potentiated the age-related increase in ME-Dif, no significant interaction was detected between the age-related increase in ME-Dif and any other baseline characteristics investigated such as sex, BMI, comorbidities, classification of hypertension, pulse rate, duration of hypertension, allergy history, previously used antihypertensive drugs, and smoking habit (Table 2). Although age-related increase in ME-Dif was observed in patients who regularly drank alcohol, no age-related pattern was observed in those who did not report regular alcohol drinking (p = 0.02) before treatment (Figure 5). However, following treatment with AZ, ME-Dif in regular alcohol drinkers decreased markedly and no significant difference was observed in these individuals versus nondrinkers (p = 0.42).

**Table 2:** Determinants of age-related increase in ME-Dif before and after AZ treatment *.

	Before AZ	After AZ
Regular alcohol drinking	<0.01	0.39
Previous beta-blocker use	0.04	0.16

* Numbers indicate p-values for age-by-baseline factors interaction.ME-Dif, difference of evening and morning SBP.

### 2.6. Safety

Incidence rate of side effects in 2,590 patients included in the safety assessment was 3.13% (81 cases). The most common side effects observed after treatment with AZ were dizziness (0.50%), headache (0.31%), palpitation (0.19),heat sensation (0.15%), and edema (0.15%). These side effects are well known associated with taking CCBs.

### 2.7. Discussion

In this study morning BP, evening BP, ME-Ave, and ME-Dif were significantly decreased following treatment with AZ. Whereas ME-Dif was found to increase with age at baseline, the increase was suppressed following treatment with AZ. Furthermore, age-related increase in ME-Dif was found potentiated by regular alcohol drinking and beta-blocker use, and the potentiation was suppressed following treatment with AZ.

AZ exhibits high affinity toward vascular tissue due to its strong lipophilicity. AZ distributes in vascular tissue for long periods, binds potently to L-type Ca-channels through the membrane approach, and thereby exerts slowly progressing and long-lasting BP-lowering effects [15,16]. In the present study AZ was confirmed significantly to decrease morning BP, evening BP, and ME-Ave and thus to exert long-lasting hypotensive activity over the 24 h dosing period.

Morning hypertension is influenced by alpha-adrenergic sympathetic nervous activity [17]. There are reports that BP tends to vary in elderly subjects due to imbalance between sympathetic alpha– and beta-adrenergic nervous activities [18,19]. Furthermore, BRS is an important control mechanism of BP that is decreased with advancing age [8,9]. Impaired BRS due to modified predominant alpha-sympathetic activity and increased arterial stiffness in the elderly may be a key physiological mechanism of increased ME-Dif [20]. AZ has been shown to ameliorate BRS [13] and to inhibit sympathetic nervous activity via vasodilation-induced baroreceptor reflex [12]. The improvement of the age-related increase in ME-Dif after treatment with AZ as observed in the present study is considered due to these known actions of this agent.

In this study the observed change of pulse rate from baseline in early morning and evening was –3.5 ± 7.8 and –3.5 ± 7.3 beats/min, respectively—both statistically significant reductions (p < 0.0001). Although it is known that some CCB agents decrease BP very rapidly resulting in enhanced sympathetic activity by baroreceptor reflex and increased pulse rate, conversely, since AZ suppresses sympathetic nervous activity this agent does not induce reflex tachycardia [12]. Thus AZ-induced decrease of pulse rate possibly contributes to improvement of BRS by this agent.

The exaggeration of the age-related increase in ME-Dif in patients who regularly drink alcohol or use beta-blockers as shown in the present study agrees well with our previous findings [7]. Other researchers have also reported increased ME-Dif caused by regular alcohol consumption [21]. Kawano *et al*. [21] showed that alcohol drinking reduces evening BP level and gradually increases morning BP level in home BP monitoring. There is a report that alcohol intake is associated with increase of ME-Dif caused by decrease of nocturnal BP and low quality of sleep associated with sympathetic nervous activity as well as increase of morning BP caused by early awaking [22].

One of the main mechanisms of morning hypertension is hyperactivity of alpha-adrenergic sympathetic nervous activity [23]. Indeed, bedtime dosing with the alpha_1_-adrenergic receptor blocker doxazosin has been reported successfully to reduce morning hypertension [24,25]. Ikeda *et al*. [26] reported that bedtime dosing of doxazosin in combination with amlodipine is useful for controlling morning BP and regression of LV hypertrophy. The exaggeration of the age-related increase in ME-Dif in patients who use beta-blockers noted in the present study is considered due to predominant alpha-sympathetic activity following blockade of beta-sympathetic neurons. Suppression of the age-related increase in ME-Dif in regular alcohol drinkers or beta-blockers users as seen in the present study may be due to inhibitory effect of AZ on sympathetic nervous activity. In the present study beta-blockers were taken in only 189 of 2,546 patients (7.4%) and it is unknown which medications exactly were used. Thus further studies with a larger number of patients are required to clarify the effects of beta-blockers intake. 

 This study contains some limitations. The protocol was designed to represent clinical practice in the "real world"; consequently patients were not blinded to treatment and no placebo comparison was made. In addition, the home BP monitors and BP measurement methods and timing of drug dosing used in the present study were not standardized. Moreover, alcohol drinking frequency of the patients was recorded only at baseline and no quantitative data on daily alcohol intake were collected. However, since the present study was prospectively investigated, and age-related increase in ME-Dif was defined by the baseline data, the results are believed reliable.

## 3. Experimental Section 

### 3.1. Study design and participants

This study followed an open, prospective, cohort design. The protocol was approved by the In-house Ethical Committee of Sankyo (presently Daiichi-Sankyo following company merger) and was based on the pharmaceutical affairs law in Japan. The protocol was submitted to and approved by the Ministry of Health, Labor and Welfare of Japan before study commencement. This study was carried out in medical institutions registered according to Good Post-marketing Study Practice in Japan.

Subjects were AZ-naïve patients with essential hypertension. Clinic BPs and morning home BPs were determined within 28 days prior to AZ dosing. Physicians in several medical institutions were asked to select and register patients at the Registration Center within 14 days of starting AZ therapy. The registration period was from May 2006 to May 2007. BP, pulse rate, and adverse events were recorded. In the present study, subjects whose evening BPs were determined prior to AZ dosing were selected for analysis. 

### 3.2. Drug administration

Based on indications and doses described in the Japanese package insert, AZ (mainly 8 or 16 mg/d) was administered at each participating physician's discretion. Neither prior treatment nor combination drugs were restricted. The standard observation period was 16 weeks. 

### 3.3. Blood pressure measurement

Since the present investigation was performed under daily clinical practice, home BP at baseline was measured by automatic device based on cuff-oscillometric method, whereas the method of that during the observation period was not strictly defined Morning home BP was determined before breakfast and AZ dosing where possible. As a gneral rule home BP was determined once at baseline and at the end of the treatment period. However, when data were collected more than once, the average value was calculated from the last two values obtained. 

### 3.4. Alcohol drinking habits

Based on medical interview at baseline, patients were classified as (1) taking alcohol almost every day; (2) taking alcohol less regularly than every day; (3) rarely taking alcohol; or (4) nondrinkers; those in group (1) were defined as "regular alcohol drinkers" in this study.

### 3.5. Data analysis

Continuous variables and categorical variables are expressed as mean (±SD) and rate (%), respectively. The time-course of changes in BP and pulse rate was analyzed by Dunnett's multiple comparisons test. BP, ME-Ave, ME-Dif, and pulse rate after AZ treatment were compared versus baseline levels by paired *t*-test. Relationships between ME-Dif and patient age before AZ dosing were assessed by regression analysis. Interactions between age and baseline characteristics of the patients were analyzed by multiple regression model. A p-value < 0.05 was defined as significant. Statistical analyses were performed using SAS System Release 8.2 (SAS Institute Inc, Cary, NC, USA).

## 4. Conclusions

Although ME-Dif increases with age, treatment with AZ can significantly suppress this pathological condition. Moreover, the age-related increase in ME-Dif is potentiated by regular alcohol drinking and beta-blocker use, and the potentiation was suppressed following treatment with AZ. AZ exerts both BRS-ameliorating effects and suppressing effects on sympathetic nervous activity. Therefore AZ is a useful agent for the treatment of early-morning hypertension, and is expected to exert beneficial effects not only on BP and pulse rate but also on risk factors for cardiovascular events. However, clinical implication of ME-Dif for cardiovascular events is not established. Whether reduction of ME-Dif leads to reduce the risk of cardiovascular events in clinical practice should be investigated in further prospective studies.
